# Metabolomic and Transcriptional Profiling of Oleuropein Bioconversion into Hydroxytyrosol during Table Olive Fermentation by *Lactiplantibacillus plantarum*

**DOI:** 10.1128/aem.02019-21

**Published:** 2022-03-22

**Authors:** Amanda Vaccalluzzo, Lisa Solieri, Davide Tagliazucchi, Alice Cattivelli, Serena Martini, Alessandra Pino, Cinzia Caggia, Cinzia L. Randazzo

**Affiliations:** a Department of Agriculture, Food and Environment, University of Catania, Catania, Italy; b Department of Life Sciences, University of Modena and Reggio Emilia, Reggio Emilia, Italy; c ProBioEtna srl, Catania, Italy; University of Helsinki

**Keywords:** β-glucosidase, brine fermentation, stress condition, gene detection, gene transcription, oleuropein

## Abstract

This study aims to elucidate the mechanisms responsible for the bioconversion of oleuropein into low-molecular-weight phenolic compounds in two selected Lactiplantibacillus plantarum strains, namely, C11C8 and F3.5, under stress brine conditions and at two different temperatures (16°C and 30°C). For this purpose, we adopted an experimental strategy that combined high-resolution mass spectrometry, *in silico* functional analysis of glycoside hydrolase family 1 (GH1)-encoding candidate genes, and gene expression studies. The oleuropein hydrolysis products and the underlying enzymatic steps were identified, and a novel putative *bgl* gene was detected, using seven strains belonging to the same species as controls. According to metabolomic analysis, a new intermediate compound (decarboxymethyl dialdehydic form of oleuropein aglycone) was revealed. In addition, strain C11C8 showed a decrease in the oleuropein content greater than that of the F3.5 strain (30% versus 15%) at a temperature of 16°C. The highest increase in hydroxytyrosol was depicted by strain C11C8 at a temperature of 30°C. PCR assays and sequencing analyses revealed that both strains possess *bglH1*, *bglH2*, and *bglH3* genes. Furthermore, a reverse transcription-PCR (RT-PCR) assay showed that *bglH3* is the only gene transcribed under all tested conditions, while *bglH2* is switched off in strain C11C8 grown at cold temperatures, and no transcription was detected for the *bglH1* gene. The *bglH3* gene encodes a 6-phospho-β-glucosidase, suggesting how phospho-β-glucosidase activity could belong to the overall metabolic strategy undertaken by L. plantarum to survive in an environment poor in free sugars, like table olives.

**IMPORTANCE** In the present study, a new candidate gene, *bglH3*, responsible for the β-glucosidase-positive phenotype in *L. plantarum* was detected, providing the basis for the future marker-assisted selection of *L. plantarum* starter strains with a β-glucosidase-positive phenotype. Furthermore, the ability of selected strains to hydrolyze oleuropein at low temperatures is important for application as starter cultures on an industrial scale.

## INTRODUCTION

The β-glucosidase enzymes are glycoside hydrolases (GHs) that catalyze the transfer of the glycosyl group between nucleophiles, enabling the release of monomers such as β-d-glucose from various disaccharides, oligosaccharides, and alkyl- and aryl-β-d-glucosides (1–3). Based on amino acid similarity and hydrophobic cluster analyses, they are classified as glycosyl hydrolase families 1 and 3, respectively (4). Glycoside hydrolase family 1 (GH1) comprises enzymes with a number of known activities, including both β-glucosidase (EC 3.2.1.21) and 6-phospho-β-glucosidase (EC 3.2.1.86). They can be isolated from plants, animals, and microorganisms. In food biotechnology, these enzymes are present in almost all microorganisms involved in the fermentation processes of plant-derived substrates, as these microbes exploit plant glycosides as a source of energy and require acid-stable β-glucosidases to release the sugar fraction at low pH (2, 5–7). In the production of table olives, the microbial β-glucosidase enzyme is responsible for the hydrolysis of the bitter compound oleuropein, releasing low-molecular-weight phenolic compounds such as hydroxytyrosol and tyrosol. It is noteworthy that strains belonging to the species Lactiplantibacillus plantarum are often used as starter cultures for table olives due to their versatility in adapting to fermentation conditions and their ability to accelerate the debittering process through β-glucosidase activity (8–10). However, little information is available on the genes encoding the β-glucosidase enzymes (9–12). For example, two different genes were reported to be responsible for β-glucosidase activity in the species *Lactiplantibacillus plantarum* (9, 12). In a recent study, 9 L. plantarum strains were proven to hydrolyze oleuropein *in vitro*, and only 5 strains possessed the *bglH* gene (lp_3525 in the WCFS1 genome) previously described as encoding a β-glucosidase enzyme (10). This genotype-phenotype inconsistency suggests that other genes may contribute to β-glucosidase activity. These genes have the potential to encode enzymes from several closely related hydrolase families, such as glycoside hydrolases and glycosyl hydrolases (13, 14). Indeed, candidate primers often identify regions where β-glucosidase genes are located, which are not closely involved in encoding the enzyme of interest. However, the ability to enzymatically degrade phenolic compounds is a strain-specific characteristic that allows the discrimination of starter strains within the same species. Genetically, discrimination at the strain level is much more difficult. Primers designed to detect β-glucosidase genes are not strain specific and therefore amplify a region where, for some strains of the same species, the candidate gene may not be present because it is located at another gene locus.

This work aims to elucidate the mechanisms responsible for oleuropein bioconversion into low-molecular-weight phenolic compounds in two selected *L. plantarum* strains previously characterized for oleuropein degradation (10), under conditions simulating the fermentation of olives in brine. For this purpose, the experimental strategy adopted integrated high-resolution mass spectrometry (HR-MS), *in silico* functional analysis of GH1 candidates, and gene expression studies. We identified oleuropein hydrolysis products, the underpinning enzymatic steps, as well as a new putative *bgl* gene responsible for the observed β-glucosidase activity under low-temperature conditions.

## RESULTS AND DISCUSSION

### Hydrolysis of oleuropein in table olive brine medium and identification of the reaction products.

**FIG 1:**
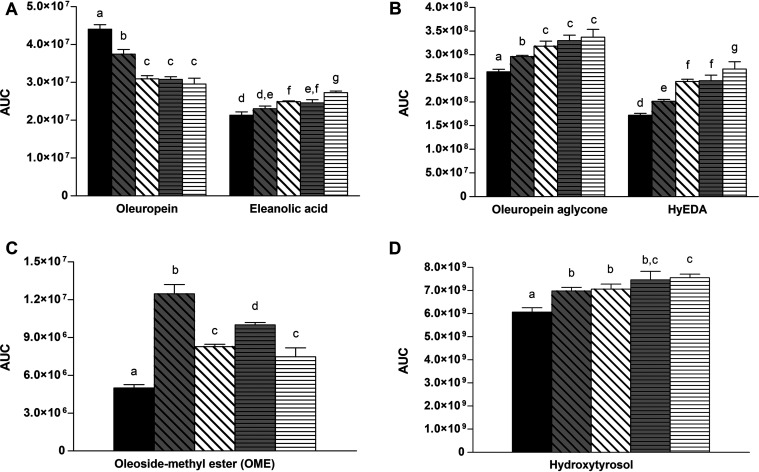
Relative quantification of phenolic and related compounds identified in control and inoculated table olive brine medium. AUCs were calculated from the extracted ion chromatograms (EICs) obtained for each compound mass-to-charge ratio (tolerance of ±3 ppm). Black bars represent the control table olive brine medium without an inoculum. Dark gray and white bars with oblique line patterns represent table olive brine media inoculated with *L. plantarum* F3.5 at 16°C and 30°C, respectively. Dark gray and white bars with horizontal line patterns represent table olive brine media inoculated with *L. plantarum* C11C8 at 16°C and 30°C, respectively. Different letters indicate significant differences (*P < *0.05). HyEDA, decarboxymethyl dialdehydic form of oleuropein aglycone. Raw data are reported in Table S2 in the supplemental material.

**FIG 2:**
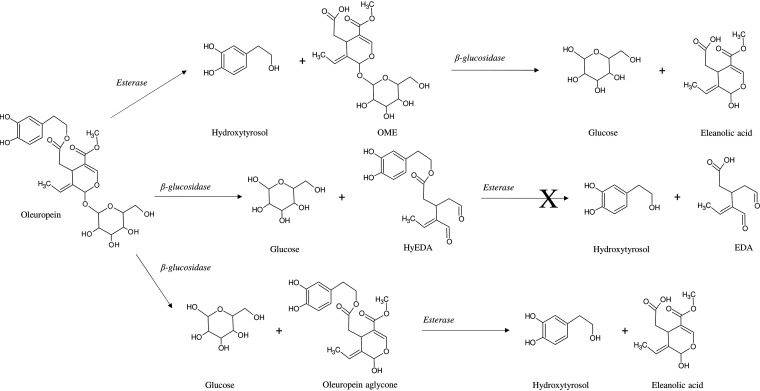
Proposed metabolic pathways for oleuropein degradation by *L. plantarum* F3.5 and C11C8. The X means that the specific pathway was not active in both strains. HyEDA, decarboxymethyl dialdehydic form of oleuropein aglycone; OME, oleoside-methyl ester; EDA, dialdehydic form of decarboxymethyl.

*L. plantarum* strains C11C8 and F3.5 were previously selected for their different aptitudes for hydrolyzing oleuropein and for the presence of the β-glucosidase gene, as reported previously by Vaccalluzzo et al. (10). However, no data are available concerning the oleuropein-degrading abilities of the *L. plantarum* strains in a brine olive-like environment. In the present study, the abilities of *L. plantarum* strains C11C8 and F3.5 were investigated by incubating the two selected strains for 72 h in table olive brine medium at two different temperatures (16°C and 30°C). The data were compared to those for a control sample, represented by table olive brine medium without inoculated strains and incubated under the same conditions as the ones reported above. Phenolic and related compounds identified by high-resolution mass spectrometry were oleuropein, oleuropein aglycone, hydroxytyrosol, oleoside-methyl ester (OME), a decarboxymethyl dialdehydic form of oleuropein aglycone (HyEDA), eleanolic acid (elenolic acid-methyl ester), and a dialdehydic form of decarboxymethyl eleanolic acid (EDA). Mass spectrometry data and relative quantification data, expressed as areas under the curve (AUCs), are reported in Tables S1 and S2 in the supplemental material, respectively. As can be observed in Fig. 1A (see also Table S2 in the supplemental material), both strains were able to hydrolyze oleuropein, as evidenced by the recorded decrease in the relative amount of this compound compared to the control. The highest decrease was detected for *L. plantarum* C11C8 after incubation in table olive brine medium at 30°C (∼33% decrease compared to the control). No significant difference in oleuropein-degrading abilities was found between the two temperatures tested for *L. plantarum* C11C8. In contrast, *L. plantarum* F3.5 showed significantly lower oleuropein hydrolytic activity at 16°C (15% decrease compared to the control) and then at 30°C (∼30% decrease compared to the control). The hydrolysis of oleuropein can involve various reaction products depending on the type of enzyme activity. Several previous studies have highlighted the ability of *L. plantarum* strains to hydrolyze oleuropein thanks to the action of bacterial β-glucosidase and esterase (9, 15–17). The β-glucosidase activity results in the hydrolysis of the glucose moiety from oleuropein, releasing oleuropein aglycone and/or HyEDA (18–20). In addition, esterase activity hydrolyzes the ester bond of oleuropein, resulting in the release of OME and hydroxytyrosol (15, 21). As shown in Fig. 1B, the relative amounts of both β-glucosidase activity products, oleuropein aglycone and Hy-EDA, increased in inoculated medium compared to the control medium (see also Table S2 in the supplemental material). The highest increase for both reaction products was found in medium inoculated with *L. plantarum* C11C8 at 30°C (28% and 56% increases for oleuropein aglycone and HyEDA, respectively). No differences were found in the relative amounts of oleuropein aglycone for *L. plantarum* C11C8 inoculated at 16°C and 30°C, while the amount of HyEDA was significantly larger in the sample incubated at 30°C. Once again, significantly larger amounts of oleuropein aglycone and HyEDA were found in *L. plantarum* F3.5 inoculated at 30°C than in medium incubated at 16°C. The concentrations of the esterase hydrolysis products OME and hydroxytyrosol also increased in inoculated medium compared to the control medium (Fig. 1C and D). The highest increase in hydroxytyrosol was found in *L. plantarum* C11C8-inoculated medium incubated at 30°C (28% increase compared to the control). For both strains, incubation at 16°C resulted in a lower level of release of hydroxytyrosol than for the sample incubated at 30°C. In contrast, the highest increase of OME was recorded in table olive brine medium incubated at 16°C for both strains. OME still contains a bound glucose moiety and can be further hydrolyzed by bacterial β-glucosidase into eleanolic acid and glucose. This pathway was further confirmed by the recorded increase in the eleanolic acid amount observed in the inoculated medium (Fig. 1A). The decrease in the concentration of OME and the increase in the amount of HyEDA observed after incubation at 30°C, compared to the medium incubated at 16°C for both strains, suggested a higher β-glucosidase level at 30°C than at 16°C. This effect was not seen in the case of oleuropein aglycone, probably because this compound was further hydrolyzed by esterase into hydroxytyrosol and eleanolic acid. Finally, no significant differences were found for EDA concentrations between the control medium and the media inoculated with the two strains (Table S2). Overall, these results confirmed the presence of oleuropein-degrading β-glucosidase and esterase activities in both tested strains. The proposed pathway of oleuropein degradation by bacterial β-glucosidase and esterase is shown in Fig. 2.

### Identification of putative *bgl* genes in the ATCC 8014 genome.

**FIG 3:**
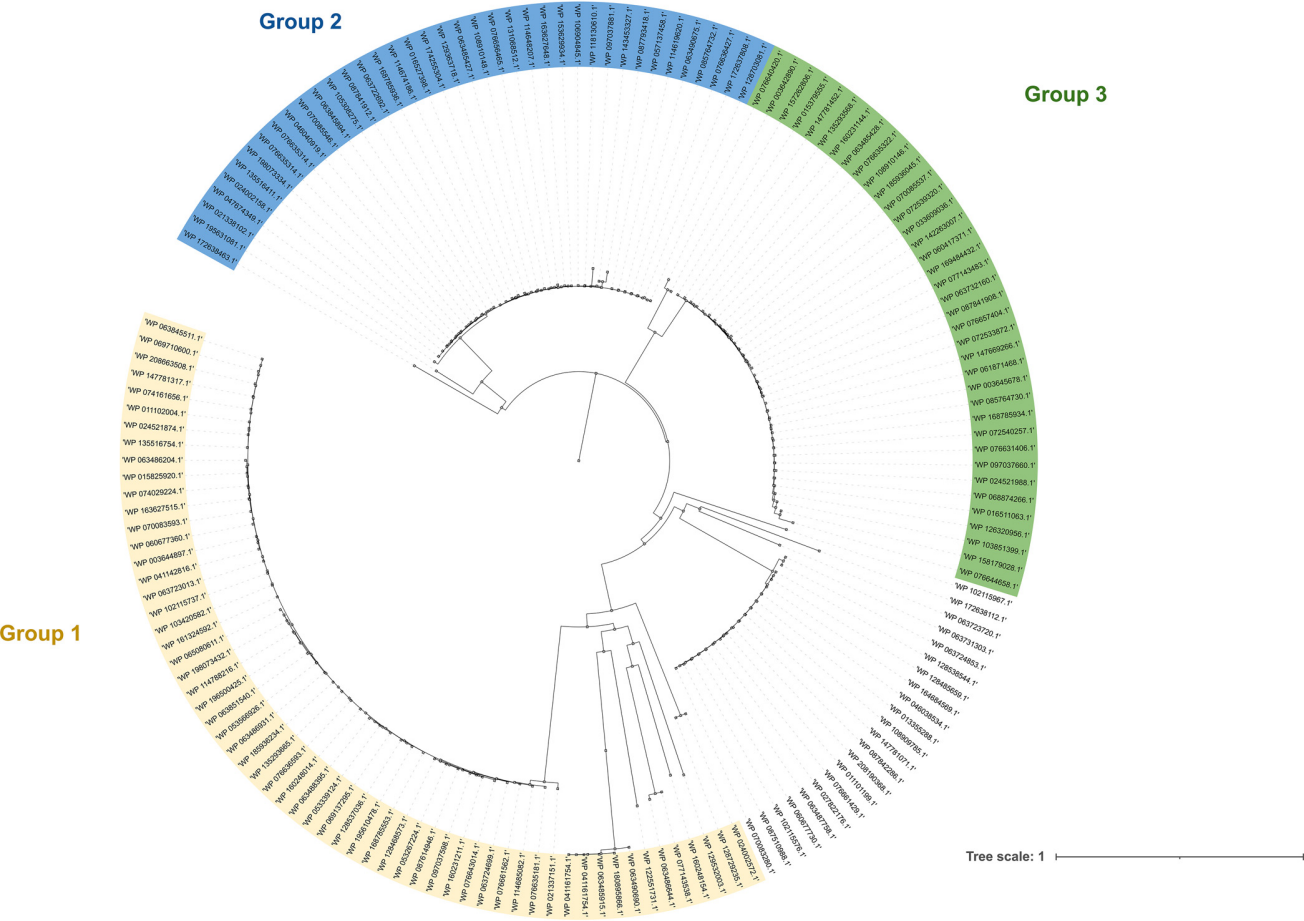
Neighbor-joining tree of *Lactiplantibacillus plantarum* glycoside hydrolase family 1 proteins (GenBank accession numbers). The analysis includes 185 RefSeq proteins (architecture identifier 10006560), which were aligned with the constraint-based alignment tool Cobalt (35). The tree was rooted using Streptococcus thermophilus (GenBank accession number WP_220023437.1) as the outgroup. The distances were computed using the Kimura 2-parameter method and are in units of numbers of base substitutions per site. The scale bar represents sequence divergence.

The proposed pathways highlight β-glucosidase as a key enzyme to degrade brine compounds and increase the concentration of hydroxytyrosol in the final product. However, different information is reported in the literature on candidate genes encoding β-glucosidases (9, 12). The most common pathway to hydrolyze β-glucosides is based on direct hydrolytic cleavage by extracellular or cell wall-associated glucosidases (EC 3.2.1.21) (22). However, several lines of evidence demonstrated that intracellular phospho-β-glucosidases (EC 3.2.1.86) are responsible for hydrolyzing C_6_-phosphorylated β-glucosides, releasing glucose-6-phosphate in several species, including Lactobacillus acidophilus (23), Streptococcus mutans (24), and Leuconostoc pseudomesenteroides (23, 25–27). β-Glucosides are generally transported by β-glucoside-specific phosphotransferase systems (PTSs), whereby the substrate is vectorially phosphorylated when taken up and subsequently cleaved by a phospho-β-glucosidase (28). In the CDD/SPARCLE database, 185 RefSeq proteins with a conserved BglB domain (COG2723) were annotated as belonging to GH1 in *L. plantarum* genomes. Clustering analysis inferred by Cobalt alignment showed that Bgl proteins grouped into three major clusters (Fig. 3). Groups 1 and 2 included proteins highly homologous to the only two Bgl proteins of *L. plantarum* with proven β-glucosidase activity, referred to as *bglH1* (12) and *bglH2* (9) and homologous to CS400_15205 and CS400_14756, respectively, in the ATCC 8014 genome (Fig. 3). While *bglH1* is annotated as a GH1 protein, *bglH2* is annotated as a 6-phospho-β-glucosidase, which differs from the other 6-phospho-β-glucosidases present in the ATCC 8014 genome for the BglB domain instead of the glyco_hydro superfamily domain (Table S3). Interestingly, a third gene in ATCC 8014, namely, *bglH3* (homologous to CS400_14770), showed a predicted BglB superfamily domain as *bglH2*, and the corresponding protein clustered into group 3 (Fig. 3). The *bglH3* gene is downstream of *bglH2* and upstream of two genes coding for a PTS permease (BglP) and a transcriptional antiterminator (BglG), respectively. This synteny resembles those described previously for the polycistronic operon *bglGFB* in Escherichia coli (29, 30), *bglP* in Bacillus subtilis (31), and *bglGPT* in *L. plantarum* strain B21 (9, 11). Recently, another polycistronic operon, including a PTS and a gene encoding 6-phospho-β-glucosidase, was found to be responsible for the utilization of plant-derived galactomannan oligosaccharides (32) and of gentiobiose and cellobiose in brewing spent grain fermentation carried out by *L. plantarum* WCFS1 (26). Based on these lines of evidence, we selected *bglH1*, *bglH2*, and *bglH3* as putative target candidates responsible for β-glucosidase activity in *L. plantarum* C11C8 and F3.5.

### Screening and phylogenetic analysis of candidate *bgl* genes in *L. plantarum* wild strains.

**FIG 4:**
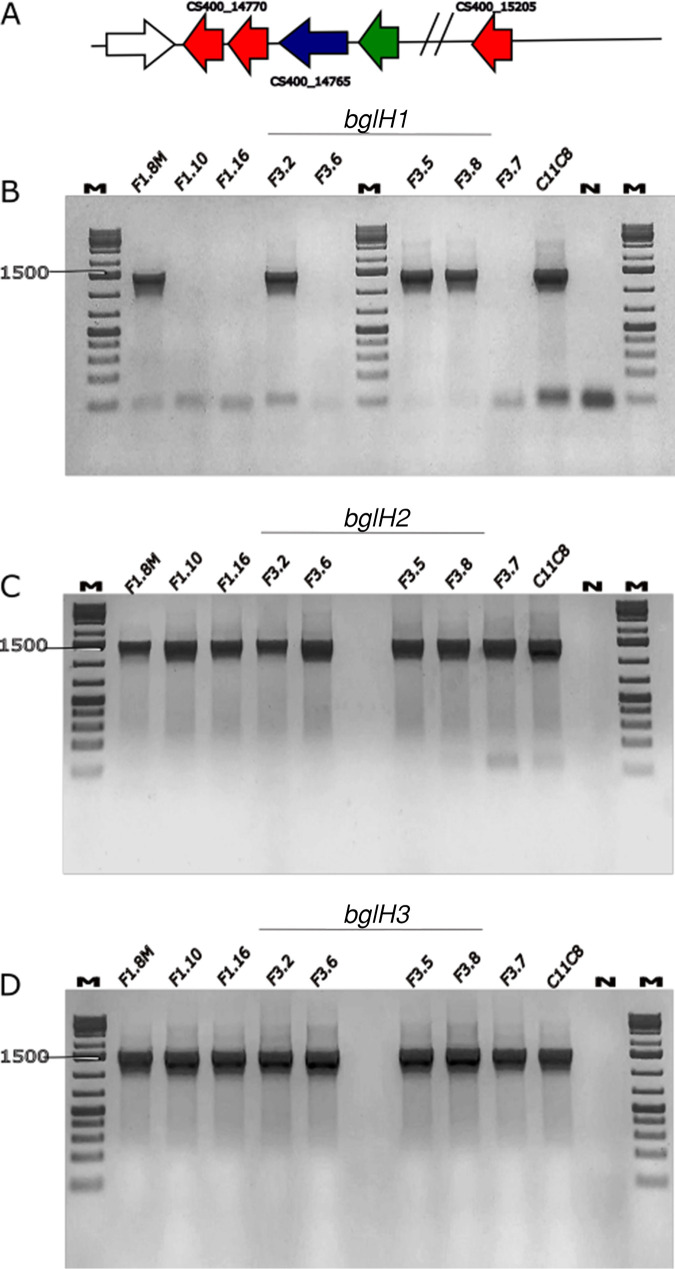
PCR assays targeting putative *bgl* genes in *Lactiplantibacillus plantarum* wild strains isolated from brine olives. (A) Cartoon representing the synteny of candidate *bgl* genes in the ATCC 8014 genome. Genes are represented as arrows and are not to scale. Red arrows indicate candidate *bgl* genes, the blue arrow represents a PTS β-glucoside transporter subunit-encoding gene, the green arrow represents the open reading frame (ORF) encoding the antitermination protein BglG, and the white arrow represents flanking genes not involved in glucoside hydrolysis. (B) PCR screening of the *bglH1* gene carried out with primer pair bglu_F/bglu_R. The expected amplicon length is 1,485 bp. (C) PCR screening of the *bglH2* gene carried out with primer pair m-bgl F/m-bgl R. The expected amplicon length is 1,490 bp. (D) PCR screening of the *bglH3* gene carried out with primer pair 14770_F/14770_R. The expected amplicon length is 1,490 bp long. N, negative control; M, molecular weight marker.

**FIG 5:**
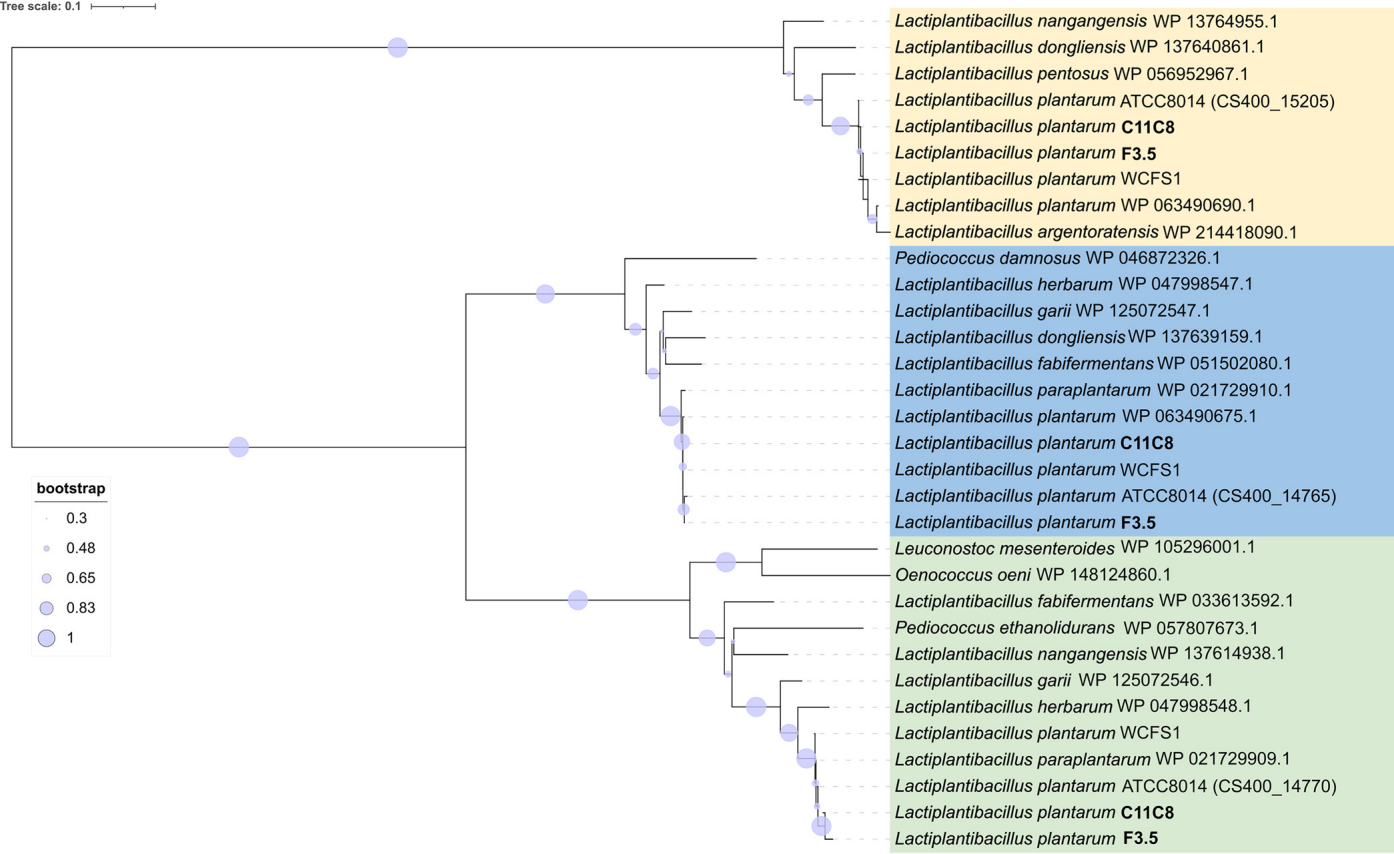
Neighbor-joining tree showing the phylogenetic positions of *bgl* candidate genes in *Lactiplantibacillus plantarum* strains C11C8 and F3.5. Amino acid sequences were aligned with Muscle. The evolutionary distances were computed using the Poisson correction method and are in units of numbers of base substitutions per site. The rate variation among sites was modeled with a gamma distribution (shape parameter of 1). Branch lengths are proportional to the numbers of nucleotide substitutions and are measured by the scale bar of sequence divergence. Bootstrap values (1,000 replicates) are shown as symbols at the nodes. Values lower than 0.3 were omitted. The analysis involved 38 amino acid sequences. All ambiguous positions were removed for each sequence pair. There were a total of 494 positions in the final data set. GenBank accession numbers are in parentheses.

A PCR assay targeting homologous genes of *bglH1*, *bglH2*, and *bglH3* was carried out on 9 *L. plantarum* strains isolated from the brine olive niche (10) and with different β-glucosidase activities against oleuropein in order to establish the gene distribution at the interstrain level. Figure 4 shows that all strains tested have the expected amplicon lengths for *bglH2* and *bglH3*, whereas 4 of the 9 strains tested did not give any PCR products with the primer pair targeting *bglH1* (12). Although we cannot exclude that single nucleotide polymorphisms (SNPs) and/or indels prevented the proper amplification of the *bglH1* gene in these wild-type strains, the data suggest that a loss or mutation of *bglH1* did not affect β-glucosidase activity against oleuropein in the set of tested strains. According to mass spectrometry analysis, we chose strains C11C8 and F3.5 to sequence the homologous genes of *bglH1*, *bglH2*, and *bglH3*. *L. plantarum* strains C11C8 and F3.5 encode two proteins that are mutually identical and highly similar to those encoded by the *bglH1* (99.89%) and *bglH2* (99.71%) genes detected in the genome of *L. plantarum* type strain ATCC 8014, whereas they differed significantly from the nucleotide sequences of the homologous *bglH3* gene (Fig. S1). Functional predictions based on analyses using the CDD/SPARCLE database revealed that the deduced amino acid sequences of both allelic variants maintained a BglB superfamily domain configuration (architecture identifier 10006560; E value of 0e+00 for both C11C8 and F3.5). However, they displayed 2 substitutions and 1 indel. Phylogenetic analysis confirmed that the genomes of strains C11C8 and F3.5 contain three genes homologous to *bglH1* (group 1), *bglH2* (group 2), and *bglH3* (group 3) (Fig. 5).

### β-Glucosidase gene expression profile in table olive brine medium.

**FIG 6:**
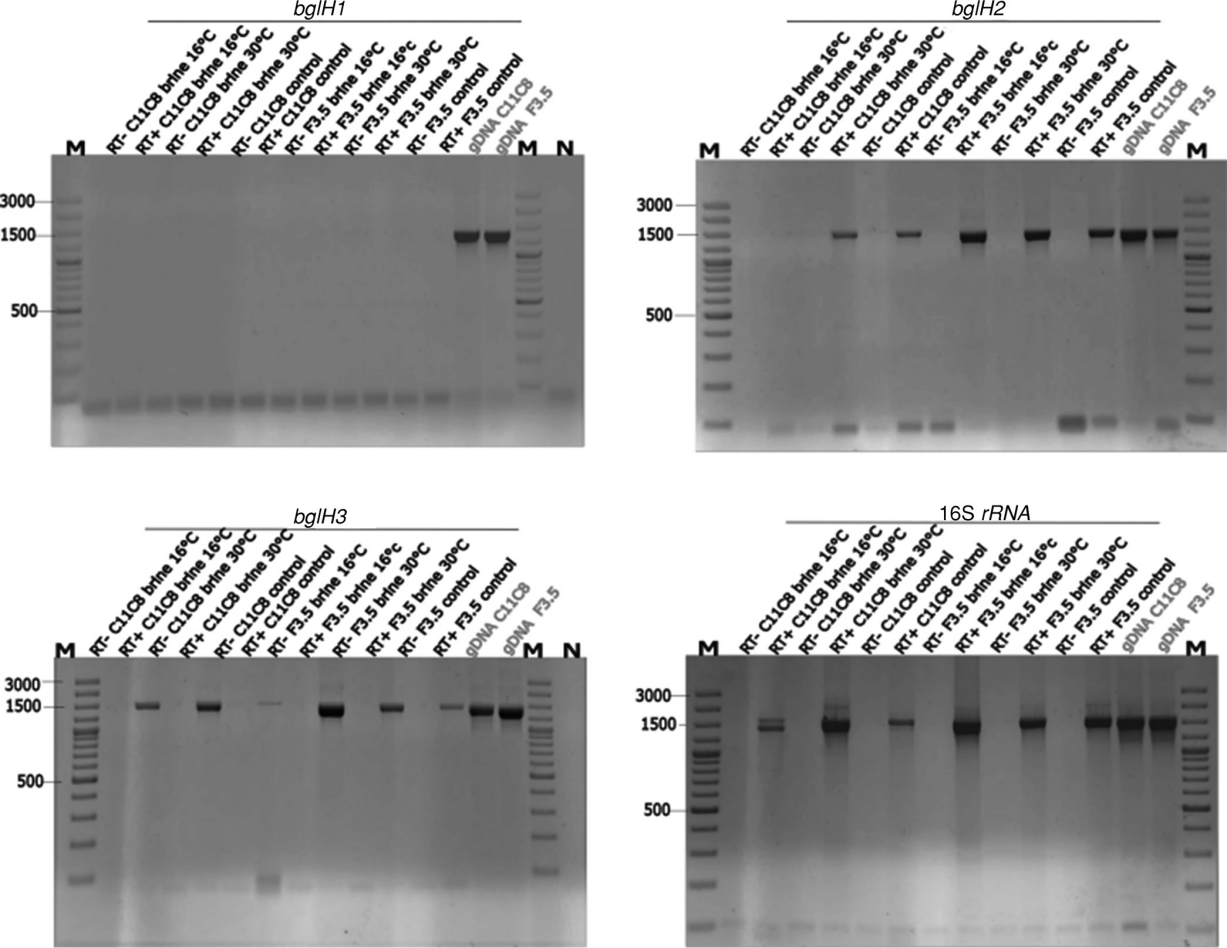
RT-PCR assays targeting putative *bgl* genes in *L. plantarum* C11C8 and F3.5. cDNA was amplified from total RNA extracted from cells growing both in table olive brine medium and under control conditions (MRS medium supplemented with 5% NaCl [pH 5.5] and glucose). Target genes are indicated near the corresponding picture of the electrophoretic gel. The expected lengths of PCR amplicons were 1,490 bp for genes homologous to *bglH1*, 1,485 bp for genes homologous to *bglH2*, and 1,485 bp for genes homologous to *bglH3*. + RT or − RT indicates with or without reverse transcriptase in the cDNA synthesis reaction, respectively. Bacterial DNA amplification was used as a positive PCR control. The 16S rRNA gene was used as a housekeeping gene. gDNA, genomic DNA; M, molecular weight marker.

In order to identify the genes responsible for β-glucosidase activity toward oleuropein in strains C11C8 and F3.5, cells were collected after 72 h of incubation in table olive brine medium at both 16°C and 30°C. Figure 6 shows that both strains did not actively transcribe the homologous *bglH1* gene regardless of the incubation temperature. Considering that C11C8 and F3.5 exhibited oleuropein degradation activity at this stage of the table olive brine assay, the lack of *bglH1* gene expression suggests that this gene may not be responsible for the observed β-glucosidase activity. Remarkably, Acebrón et al. (32) cloned and heterologously expressed this gene in Escherichia coli and proved that the resulting recombinant enzyme has galactosidase but not glucosidase activity.

A reverse transcription-PCR (RT-PCR) assay targeting the homologous *bglH2* gene showed that F3.5 actively transcribed this gene at both 16°C and 30°C, while C11C8 switched off gene expression under cold conditions (Fig. 6). Comparison of the gene expression profiles with the mass spectrometry data suggested that in strain C11C8, the *bglH2* gene might contribute to β-glucosidase activity toward oleuropein at 30°C but not at 16°C. In contrast, *L. plantarum* strain C11C8 increased the transcription signal of the *bglH3* gene at both 16°C and 30°C when grown in olive brine medium (Fig. 6). Further quantitative PCR (qPCR) studies should be performed to quantitatively confirm this gene transcription profile, but overall, these data have demonstrated that, different from previous reports (12), the *bglH1* gene is not involved in the hydrolysis of oleuropein to aglycone under table olive brine conditions and that a new candidate gene, namely, *bglH3*, could be related to β-glucosidase activity at 16°C, in addition to *bglH2* (9).

### Conclusions.

The β-glucosidase enzyme is involved in several biotechnological processes. Until now, only a few studies have investigated the mechanisms of β-glycoside hydrolysis in strains belonging to the species *Lactiplantibacillus plantarum*, which are generally recognized as safe (GRAS). In this study, mass spectrometry, *in silico* protein analysis, and gene expression profile analysis revealed that oleuropein bioconversion into a hydroxytyrosol compound is a complex phenotype, occurring through at least three different metabolic pathways and involving the concerted action of both β-glucosidase and esterase enzymatic activities. Although further quantitative analyses are required to corroborate these data, this is the first study to identify specific metabolic pathways in *L. plantarum* that mediate the enzymatic degradation of oleuropein by analysis of changes in specific metabolites. We also demonstrated that the set of *bgl* genes responsible for β-glucosidase activity could be more complicated than what was previously supposed and identified a new candidate gene encoding phospho-β-glucosidase, namely, *bglH3*, for future functional studies. Interestingly, 6-phospho-β-glucosidase activity links the metabolism of β-glucosides with the requirement for *L. plantarum* strains to survive under glucose depletion in table olive brine and could represent a selective advantage that allows *L. plantarum* to use oleuropein as a source of glucose. Similarly, *L. plantarum* and Leuconostoc pseudomesenteroides grown on brewing spent grain showed increased expression of 6-phospho-β-glucidase-encoding genes allocated in their respective operons along with a substrate-specific PTS as the main metabolic pathway for carbon catabolism during plant-based fermentation (27). Remarkably, strains C11C8 and F3.5 have different patterns of metabolites at 16°C, and their genomes harbor slightly different variants of the *bglH3* gene. Further experiments are required to experimentally validate this relationship; different alleles at this locus could be responsible for the differential β-glucosidase activity under low-temperature conditions. The data collected in this study pointed out the importance of genes encoding phospho-β-glycosidase in debittering fermented table olives and enriching this fermented food in hydroxytyrosol precursors. This information could have important practical implications for table olive brine fermentation and could drive future selection criteria for new oleuropein-degrading *L. plantarum* starter cultures.

## MATERIALS AND METHODS

### Bacterial strains and culture conditions.

Two oleuropein-degrading *Lactiplantibacillus plantarum* strains, namely, F3.5 and C11C8, were considered in this study. In addition, seven *L. plantarum* strains (F1.8M, F1.10, F1.16, F3.2, F3.3, F3.6, F3.7, and F3.8) were used as controls for the genomic detection of the candidate β-glucosidase genes. All strains belong to the Collection of the Department of Agriculture, Food, and Environment (Di3a), University of Catania (Italy), and were propagated statically in de Man-Rogosa-Sharpe (MRS) broth (Oxoid, Basingstoke, Hampshire, UK) under aerobic conditions or on MRS agar plates (1.5% [wt/vol]) under anaerobic conditions by using an Anaerogen kit (Oxoid, Milan, Italy) at 30°C. The strains were maintained on soft agar (0.7% [wt/vol]) MRS medium for the duration of the experiments.

### Olive brine fermentation assay.

Bacterial cells were harvested by centrifugation (10,000 rpm for 10 min at 4°C) in the stationary phase (optical density at 600 nm [OD_600_] of 1.7 to 2.5), washed in a saline solution (0.9% NaCl), and used to inoculate filtered (0.22 μm) table olive brine medium (brine solution at 7% NaCl of cracked Nocellara Etnea table olives after 120 days of fermentation) at a final concentration of 9 log CFU/mL. The two strains were incubated at a temperature of 16°C for 72 h in order to simulate the stressful conditions of Sicilian table olive fermentation. In addition, a temperature of 30°C, ideal for the growth of the two tested strains and for β-glucosidase activity, was used as the control temperature.

After 72 h of incubation at both 30°C and at 16°C, cells at a density of 8 log CFU/mL were collected by centrifugation (10,000 rpm for 5 min) and used for RNA extraction, while the supernatant was used for the high-resolution mass spectrometry (HR-MS) analysis. Table olive brine medium without an inoculum was used as a control. The assay was carried out in triplicate.

### High-resolution mass spectrometry analysis.

Two milliliters of the collected supernatants was freeze-dried, and the obtained powder was resuspended in 2 mL of dimethyl sulfoxide (Merck KGaA, Darmstadt, Germany). After centrifugation (10,000 rpm for 20 min at 4°C), samples were filtered at 0.22 μm to remove insoluble material and subjected to ultrahigh-performance liquid chromatography (UHPLC)/HR-MS analysis for the identification of phenolic and related compounds and relative quantification. For each sample, 10 μL was injected into a UHPLC Ultimate 3000 separation module outfitted with a C_18_ column (Acquity UHPLC high-strength silica C_18_ reversed phase, 2.1 by 100 mm, 1.8-μm particle size; Waters, Milan, Italy). MS and tandem mass spectrometry (MS^2^) experiments were carried out on a high-resolution Q Exactive hybrid quadrupole-orbitrap mass spectrometer (Thermo Scientific, San Jose, CA, USA). The flow rate was fixed at 0.3 mL/min. The chromatographic conditions and the MS and MS^2^ parameters were fully described previously by Martini et al. (33). The relative quantification of phenolic and related compounds was performed by integrating the area under the curve (AUC) using the Genesis algorithm function in the Thermo Xcalibur quantitative browser. AUCs were calculated from the extracted ion chromatograms (EICs) achieved for each compound mass-to-charge ratio with the tolerance set at ±3 ppm.

### *In silico* analysis and primer design for the β-glucosidase gene.

Amino acid sequences used in the present study were extracted from the RefSeq CDD/SPARCLE database (architecture identifier 10006560) (34). The sequences were selected in an effort to prepare a representative data set of *L. plantarum* GH1 members as possible. Multiple alignments of protein sequences were performed using the Cobalt tool (35) with default settings, and a phylogenetic tree was built using the Kimura 2-parameter (K2P) model and the neighbor-joining (NJ) method. The tree was visualized using Interactive Tree of Life (ITOL) (36) and rooted with the outgroup reference species Streptococcus thermophilus. Experimentally validated *bgl* genes were retrieved from the Brenda database (37).

### Bacterial DNA extraction and PCR gene-specific screening.

Bacterial DNA was extracted from a culture incubated overnight at 37°C according to the protocol proposed previously by Gala et al. (38). The extracted DNA was suspended in Tris-EDTA (TE) buffer (10 mmol/L Tris-HCl, 1 mmol/L EDTA [pH 8.0]) and quantified spectrophotometrically using a Nanodrop Nd 1000 system (Nanodrop Technologies, Wilmington, DE, USA). DNA quality was confirmed by electrophoretic running on 0.8% (wt/vol) agarose gels in 0.5× Tris-borate-EDTA (TBE) buffer (45.0 mmol/L Tris-borate and 1.0 mmol/L EDTA [pH 8.0]) and subsequent UV visualization after staining with ethidium bromide (0.5 μg/mL). All primers used in this study are detailed in Table 1. Primer pair 14770_F/14770_R was designed with Primer 3 software (39), using *L. plantarum* ATCC 8014 as the reference genome (BioProject accession number PRJNA415899). All PCRs were performed in 20-μL volumes with 0.4 to 0.8 μmol/L of each primer, 0.1 U of Dream *Taq* (Thermo Scientific, Waltham, MA, USA), 0.2 mmol/L of deoxynucleoside triphosphates (dNTPs) (Thermo Scientific, Waltham, MA, USA), 2 μL of 1× Dream *Taq* buffer (Thermo Scientific, Waltham, MA, USA), and 50 ng of bacterial DNA. PCRs were performed in a T100 thermal cycler (Bio-Rad, Hercules, CA, USA), and the thermal conditions are detailed in Table 1.

**TABLE 1:** Primers and cycling conditions used in this study

Target	Primer	Primer sequence	Primer positions	Amplification program	Expected length (bp)	Reference
*bglH1*	bglu_F	5′-GTGACTATGGTAGAGTTTCC-3′		95°C for 5 min; 35 cycles of 95°C for 30 s, 54°C for 30 s, and 72°C for 1 min); and 72°C for 10 min	1,485	12
	bglu_R	5′-TCAAAACCCATTCCGTTCCCCA-3′				

*bglH2*	m-bgl F	5′-TGATTATACTTGTTGTAAGGGCTATCATTATTAGCTAACT-3′	177–216	95°C for 5 min; 35 cycles of 95°C for 30 s, 62°C for 30 s, and 72°C for 1 min; and 72°C for 10 min	1,485	8
	m-bgl R	5′-CATTATGCTTAATCACATCTTGATACCAGTAGAACGACTT-3′	1662–1622			

*bglH3*	14770_F	5′-CAACTGGCTTTCCAAAGAAC-3′		95°C for 5 min; 35 cycles of 95°C for 30 s, 56°C for 30 s, and 72°C for 1 min; and 72°C for 10 min	1,490	This work
	14770_R	5′-CTGAATATCAATTATTAATAACTATCCCAA-3′				

16S rRNA	27F	5′-CTGGGATCCATTTACTCGAGAGTTTGATCCTGGCTCAG-3′		95°C for 5 min; 30 cycles of 95°C for 1 min, 58°C for 2.5 min, and 72°C for 2 min; and 72°C for 5 min	1,500	22
	1490R	5′-GGTTCCCCTAAGCTTACCTTGTTACGACTTC-3′				

### Sequencing and phylogenetic analysis.

PCR products were purified with a DNA Clean & Concentrator-5 kit (Zymo Research, Orange, CA, USA) and sequenced on both strands using a DNA Sanger dideoxy sequencing process. Sequences were assembled in DNAStar (DNAStar, Inc., Madison, WI, USA) and trimmed on both ends to remove primer sequences. Alignment was carried out with the Muscle program (40) using MEGA X software (41), and the resulting alignment was subjected to DNA substitution model analysis to select the best-fitting model. Phylogenetic relationships were inferred using the Poisson correction model (42) and the neighbor-joining method. Among sites, rate variation was modeled by a gamma distribution (shape parameter of 1). Bootstrap support values were obtained from 1,000 random resamplings. Alignments were visualized using Jalview v2.11 (41, 43), while trees were constructed using iTOL v5.2 as reported above.

### RNA extraction and RT-PCR assays.

Bacterial cells from the table olive brine assay were submitted to RNA extraction as previously reported (44). Briefly, cells were washed twice with diethyl pyrocarbonate [DEPC]-treated TE buffer (100 mmol/L Tris-HCl, 50 mmol/L EDTA [pH 8.0]), and cell pellets were maintained at −80°C until they were thawed with 1 mL of Tri reagent using the Zymo Direct-zol RNA MiniPrep kit (Zymo Research, Irvine, CA). Mechanical lysis was performed using a Vortex Genie 2 instrument (Mo Bio Laboratories) for two rounds of 20 min at the highest speed alternating with 3 min on ice. The quantity of total RNA was measured spectrophotometrically using a Nanodrop Nd 1000 system (Nanodrop Technologies, Wilmington, DE, USA), while the integrity was checked by denaturing gel electrophoresis on a 0.9% (wt/vol) agarose gel with formaldehyde (10 mL of 10× morpholinepropanesulfonic acid [MOPS] running buffer) and 18 mL of 37% formaldehyde (12 mol/L) in pH 7.0 1× MOPS running buffer (0.4 mol/L MOPS, 1 mol/L sodium acetate, and 0.01 mol/L EDTA) after RNA treatment at 65°C for 10 min. PCRs were carried out as reported above but using cDNA as the template instead of bacterial DNA. The 16S rRNA gene was used as a reference gene and amplified according to methods described previously by Tagliazucchi et al. (45). Cells grown in MRS medium at pH 5.5 supplemented with 5% (wt/vol) NaCl were used as controls.

### Statistical analysis.

Data are reported as means ± standard deviations from three analytical replicates for each prepared sample. Multiple comparisons among data were performed by univariate analysis of variance (ANOVA) with Tukey’s *post hoc* test using GraphPad Prism 6.0 (GraphPad Software, San Diego, CA, USA). The differences were considered significant when the *P* value was <0.05.

### Data availability.

The nucleotide sequences obtained in this study were deposited in GenBank under accession numbers OK127686 to OK127691.
