# Supplementary material for: Metabolomic and Transcriptional Profiling of Oleuropein Bioconversion into Hydroxytyrosol during Table Olive Fermentation by Lactiplantibacillus plantarum
Source: Appl Environ Microbiol. 2022 Mar 22;88(6):e02019-21. doi: 10.1128/aem.02019-21 (PMC8939334; doi:10.1128/aem.02019-21)
Supplement: Supplemental file 1 — Tables S1 to S3 and Fig. 1. Download aem.02019-21-s0001.pdf, PDF file, 1.0 MB [file aem.02019-21-s0001.pdf]

Metabolomic and transcriptional profiling of oleuropein bioconversion into hydroxytyrosol during table olive fermentation by Lactiplantibacillus plantarum

Amanda Vaccalluzzo[1], Lisa Solieri[2]*, Davide Tagliazucchi[2], Alice Cattivelli[2], Serena Martini[2], Alessandra Pino[1,3], Cinzia Caggia[1,3], Cinzia L. Randazzo[1,3]*

[1]Department of Agricultural, Food and Environment, University of Catania, Catania, Italy

[2]Department of Life Sciences, University of Modena and Reggio Emilia, Reggio Emilia, Italy

[3]ProBioEtna srl, Spin-off of University of Catania, Catania, Italy

*Corresponding authors: Lisa Solieri (lisa.solieri@unimore.it) and Cinzia L. Randazzo (cranda@unict.it)

Supplementary Material

Supplementary Table S1. High-resolution mass spectrometry data for phenolic and related compounds identified in control and inoculated table olive brine medium.

Compound	*Rt*	*Formula*	*Observed m/z*	*Calculated m/z*	*ppm*	*MS² fragment ions*
Hydroxytyrosol	5.19	$C_8H_{10}O_3$	153.0557	153.0557	0.0	123.0440
Eleanolic acid	7.50	$C_{11}H_{14}O_6$	241.0716	241.0718	-0.83	209.0452; 165.0548; 137.0597
EDA	7.51	$C_9H_{12}O_4$	183.0659	183.0663	-2.18	139.0754
OME	10.57	$C_{17}H_{24}O_6$	403.1248	403.1246	0.50	223.0617
Oleuropein	14.47	$C_{25}H_{32}O_{13}$	539.1774	539.1770	0.74	307.0826; 275.0953; 241.0717
Oleuropein aglycone	16.76	$C_{19}H_{22}O_8$	377.1247	377.1242	1.33	345.0981; 275.0943
HyEDA	17.04	$C_{17}H_{20}O_6$	319.1189	319.1187	0.63	183.0656; 139.0754

Abbreviation are: Rt: retention time; EDA: dialdehydic form of decarboxymethyl elenolic acid; OME: oleoside-methyl ester; HyEDA: decarboxymethyl dialdehydic form of oleuropein aglycone

Supplementary Table S2. Relative quantification data, expressed as AUC, for phenolic and related compounds identified in control and inoculated table olive brine medium.

Compound	*Control*	*F3.5 16 °C*	*F3.5 30 °C*	*C11C8 16 °C*	*C11C8 30 °C*
Hydroxytyrosol	$6.073x10^9 \pm 1.834x10^8$	$6.981x10^9 \pm 1.579x10^8$	$7.063x10^9 \pm 2.180x10^8$	$7.463x10^9 \pm 30.643x10^8$	$7.559x10^7 \pm 1.533x10^8$
Eleanolic acid	$2.131x10^7 \pm 8.858x10^5$	$2.301x10^7 \pm 7.153x10^5$	$2.488x10^7 \pm 2.103x10^5$	$2.457x10^7 \pm 8.251x10^5$	$2.729x10^7 \pm 4.128x10^5$
EDA	$4.250x10^8 \pm 5.960x10^6$	$4.250x10^8 \pm 4.214x10^6$	$4.149x10^8 \pm 3.966x10^6$	$4.173x10^8 \pm 4.113x10^6$	$4.112x10^8 \pm 7.4286x10^6$
OME	$4.996x10^6 \pm 2.550x10^5$	$1.245x10^7 \pm 7.375x10^6$	$8.271x10^6 \pm 1.887x10^5$	$9.911x10^6 \pm 1.931x10^5$	$7.472x10^6 \pm 7.024x10^5$
Oleuropein	$4.406x10^7 \pm 1.176x10^6$	$3.743x10^7 \pm 1.2426x10^6$	$3.092x10^7 \pm 8.409x10^5$	$3.076x10^7 \pm 7.233x10^5$	$2.957x10^7 \pm 1.541x10^6$
Oleuropein aglycone	$2.638x10^8 \pm 5.437x10^6$	$2.961x10^8 \pm 2.628x10^6$	$3.181x10^8 \pm 1.081x10^7$	$3.297x10^8 \pm 1.176x10^7$	$3.373x10^7 \pm 1.653x10^6$
HyEDA	$1.723x10^8 \pm 4.022x10^6$	$2.016x10^8 \pm 4.019x10^6$	$2.435x10^8 \pm 4.751x10^6$	$2.450x10^8 \pm 1.171x10^7$	$2.697x10^8 \pm 1.547x10^7$

Abbreviation are: AUC: under the curve; EDA: dialdehydic form of decarboxymethyl elenolic acid; OME: oleoside-methyl ester; HyEDA: decarboxymethyl dialdehydic form of oleuropein aglycon

Supplementary Table S3. Glycoside hydrolase genes annotated in ATCC8014 genome. Conserved domains were scored with CDD/SPARCLE database.

Locus tag (ATCC8014)	Coordinates (nt)	Product	Conserved domain	Accession	Lenght (aa)	Size (kDa)	References
CS400_01860	3391..392.832	glycoside hydrolase family 1 protein			478	54.9	nr
CS400_06130	1.264.972..1.266.444	6-phospho-beta-glucosidase	Glyco_hydro super family	cl23725	490	55.9	nr
CS400_11660	2.377.496..2.378.938	6-phospho-beta-glucosidase	Glyco_hydro super family	cl23725	480	54.7	nr
CS400_11665	2.378.956..2.380.395	6-phospho-beta-glucosidase	Glyco_hydro super family	cl23725	479	54.9	nr
CS400_13105	2.699.923..2.701.356	6-phospho-beta-glucosidase	Glyco_hydro super family	cl23725	477	54.4	(1)
CS400_14765	3.039.732..3.041.174	6-phospho-beta-glucosidase	BglB super family	cl40694	480	54.6	(2)
CS400_14770	3.041.263..3.042.826	6-phospho-beta-glucosidase	BglB super family	cl40694	487	55.3	nr
CS400_03890	805.939..807.441	glycoside hydrolase family 1 protein	BglB	COG2723	500	57.9	nr
CS400_12630	2.588.758..2.590.140	glycoside hydrolase family 1 protein	BglB	COG2723	460	53.4	nr
CS400_14705	3.024.868..3.026.328	glycoside hydrolase family 1 protein	BglB	COG2723	486	55.8	nr
CS400_15205	3.137.467..3.138.852	glycoside hydrolase family 1 protein	BglB	COG2723	461	52.8	(3)

Supplementary Figure S1. Multiple sequence alignment of amino acid sequences of CS400_14770 of *Lactiplantibacillus plantarum* C11C8 e F3.5 with reference strain ATCC8014. Amino acid sequences were aligned using Muscle program (4) in MEGA X software (5) and the resulting alignment was visualized using JalView v2.11 (6).

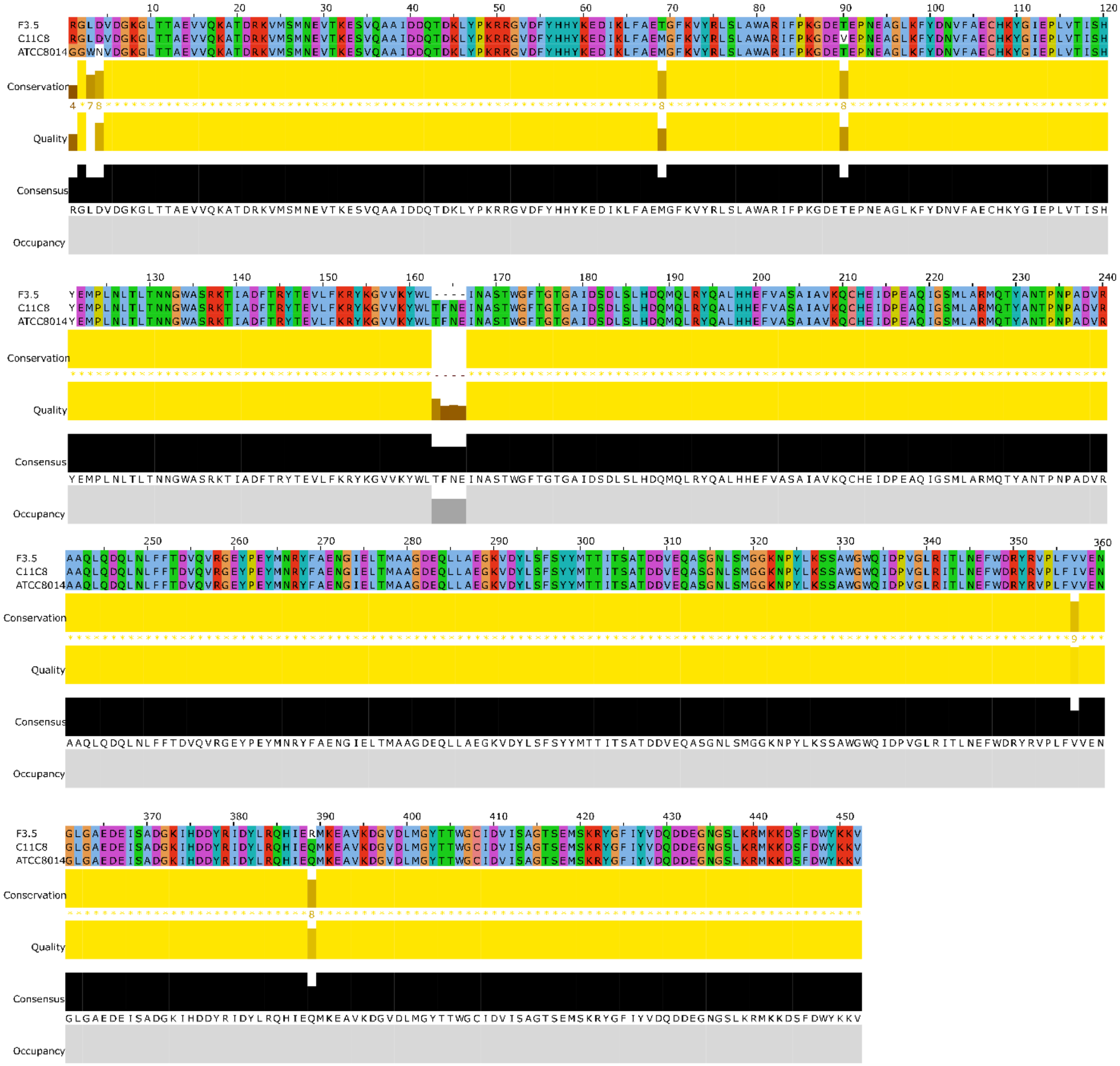

References

1. Acin-Albiac M, Filannino P, Arora K, Da Ros A, Gobbetti M, Di Cagno R. 2021. Role of lactic acid bacteria phospho-β-glucosidases during the fermentation of cereal by-products. Foods. 10:1–14.
2. Zago M, Lanza B, Rossetti L, Muzzalupo I, Carminati D, Giraffa G. 2013. Selection of Lactobacillus plantarum strains to use as starters in fermented table olives: Oleuropeinase activity and phage sensitivity. Food Microbiology. 34:81–7.
3. Spano G, Rinaldi A, Ugliano M, Moio L, Beneduce L, Massa S. 2005. A β-glucosidase

gene isolated from wine Lactobacillus plantarum is regulated by abiotic stresses. Journal of Applied Microbiology. 98:855–61.

4. Edgar RC. 2004. MUSCLE: Multiple sequence alignment with high accuracy and high throughput. Nucleic Acids Research. 32:1792–7.

5. Patel H, Kumar AK, Shah A. 2018. Purification and characterization of novel bi-functional GH3 family β-xylosidase/β-glucosidase from Aspergillus niger ADH-11. International Journal of Biological Macromolecules. 1;109:1260–9.

6. Waterhouse AM, Procter JB, Martin DMA, Clamp M, Barton GJ. 2009. Jalview Version 2-A multiple sequence alignment editor and analysis workbench. Bioinformatics. 25:1189–91.
